# Stent platforms anno 2015: is there still a place for bare metal stents at the front line?

**DOI:** 10.1007/s12471-015-0657-x

**Published:** 2015-01-27

**Authors:** Jors Nicolaas van der Sijde, Evelyn Regar

**Affiliations:** Thoraxcenter, Erasmus MC, Bd 585, ’s-Gravendijkwal 230, 3015 CE Rotterdam, The Netherlands

Coronary stenting is the preferred treatment for symptomatic stenotic coronary artery disease since the mid-1990s. Early limitations of bare metal stents (BMS), namely (sub)acute stent thrombosis, were relatively quickly mastered by recognising the importance of optimal stent expansion and the introduction of dual antiplatelet therapy. The main limitation affecting the long-term outcome of BMS, namely excessive neointima formation causing stent restenosis, was way more challenging. The quest for solving this problem triggered the proposal of a variety of strategies including intravascular ultrasound-guided stent optimisation, rota-stenting, brachytherapy and local and systemic pharmacotherapy. Finally, drug-eluting stents (DES) were successfully developed to overcome this problem at the beginning of the millennium. Restenosis rates have dropped dramatically since and DES are superior to BMS in almost every situation [1]. This had established DES as standard of care. While DES do not induce the fast neointimal growth that leads to restenosis in BMS typically within the first 6 months after implantation, we recognised a new enemy: (very) late stent failure beyond 12 months, caused by development of early neoatherosclerosis within the DES on the one hand [2] and impaired healing on the other hand. Against this background, the quest for improving stent technology is ongoing. The concept of BMS, where both neoatherosclerosis and impaired healing play a less apparent clinical role, is again of interest. Especially as recent advances in material and production methods have created new possibilities in stent design and allow for the production of very thin metal stents struts.

This is of note, as the pivotal role of stent design and geometry on neointima formation and stent thrombosis is well recognised. More specifically, experimental and clinical studies have confirmed again and again the importance of reducing stent strut thickness, with thinner struts, eliciting less restenosis than thicker struts in both BMS and DES [3]. Thicker struts increase blood flow separation, stagnation and reattachment which can ultimately lead to an increased rate of stent thrombosis [4].

In the current issue of the Netherlands Heart Journal, Suttorp et al. present a result of today’s quest for optimised stent design and deserve to be congratulated on their work. The SOLSTICE registry [5] included a series of patients treated in four Benelux centres with the SolarFlex BMS (DISA Vascular, Cape Town, South Africa, CE approval February 2014), a balloon-expandable Cobalt Cromium stent with remarkable thin struts of 65 µm. The clinical outcome up to 6 months is encouraging with a major adverse cardiac event (MACE) rate of 5.8 %. SOLSTICE is a registry, with all its advantages and disadvantages. The registry focuses on practical aspects such as device success. The patient population is more likely to reflect a real-world cathlab population with a considerable amount of patients with acute coronary syndromes (47.1 %) and diabetes (29 %) being included. However, relatively simple lesions were selected for treatment as reflected by the use of single stent (as per protocol), stent diameter (mean 3.1 mm), stent length (mean 17.4 mm) and low grade lesion angulation and tortuosity. Follow-up is clinical and extends up to 6 months, which is known to be the period when the peak of neointimal growth after BMS implantation has been reached [6]. Importantly, no follow-up angiography has been performed, which is in line with the contemporary focus on clinical outcome (as opposed to the focus on angiographic outcome in the early days of BMS) and the notion that follow-up angiography in itself can trigger revascularisation events. This ‘oculo-stenotic reflex’ has been shown to potentially double the revascularisation rate. When comparing MACE and target-vessel revascularisation rates of contemporary studies with clinical follow-up of BMS studies from the past with mandatory angiographic follow-up, this has to be taken into account. The SOLSTICE registry reports a target-vessel failure rate of 5 % which is remarkably low for BMS. Having said that, it would have been particularly interesting to gain insights into the pathophysiological mechanism of the stent failure cases which could further increase the knowledge about the role of strut thickness in vascular response and how to avoid unfavourable outcome.

What can we expect in 2015—will we be back to the future with the renaissance of sophisticated BMS? Certainly, current developments in stent design make for an interesting time in the catheterisation laboratory. There is innovation, allowing the realisation of more challenging stent designs, both in stent geometry as well as material characteristics. We will be able to offer our patients ‘stents to stay’ and bioresorbable ‘stents to disappear’, a concept that has only just begun. Carefully designed mechanistic studies and clinical observations will be needed to determine the role and most appropriate indications for their use.
